# Ultrasonographic Changes of Abdominal Muscles in Subjects with and without Chronic Low Back Pain

**DOI:** 10.3390/healthcare10010123

**Published:** 2022-01-08

**Authors:** Iria Da Cuña-Carrera, Alejandra Alonso-Calvete, Eva M. Lantarón-Caeiro, Mercedes Soto-González

**Affiliations:** Facultade de Fisioterapia, Universidade de Vigo, Campus A Xunqueira s/n, 36005 Pontevedra, Spain; iriadc@uvigo.es (I.D.C.-C.); alejalonso@uvigo.es (A.A.-C.); m.soto@uvigo.es (M.S.-G.)

**Keywords:** abdominal muscles, ultrasonography, low back pain, physiotherapy

## Abstract

Chronic low back pain (CLBP) is a prevalent disfunction in the spine, affecting both women and men. The implication of the abdominal muscles in this disfunction has been studied, including wrong breathing patterns or inactivity of this area. However, there is a lack of studies examining changes in thickness of abdominal with ultrasonography. Thus, the aim of this study is to analyze the differences in the thickness of abdominal muscles at rest and during breathing between subjects with and without CLBP. A total of 72 subjects were divided in two groups: participants with CLBP (*n* = 36) and participants without CLBP (*n* = 36). In both groups, the thickness of the four abdominal muscles was measured and compared at rest and during breathing with ultrasonography. In TrA and IO there were no significant differences between groups, but those subjects with CLBP increased the muscle thickness more than participants without pain during breathing. In EO there were no differences in muscle thickness between groups and between rest and breathing. In RA, subjects with CLBP showed less muscle thickness than subjects without pain during breathing, but no changes were found at rest. In conclusion, the deepest abdominal muscles, TrA and IO, appear to increase their thickness and RA appear to decrease more in subjects with CLBP, in comparison with healthy participants.

## 1. Introduction

Low back pain is considered as one of the main disorders involving the spine, and one of the most prevalent in adults of both sexes, with a higher prevalence in women [1,2]. Specifically, chronic low back pain (CLBP) has been described as one of the major reasons for medical consultation all over the world, with a high cost for health provider systems [3,4]. Several treatments have been described for CLBP, including pharmacology [5], surgery [6] or ergonomic education [7], depending on the severity of the disorder. However, the current evidence suggests other approaches such as manual therapy [7], therapeutic exercise [8,9] and reeducation of the breathing patterns [10], with greater benefits addressing CLBP. In this sense, the importance of the abdominal muscles in CLBP has been clearly demonstrated in prior research [11,12], since the impact of these muscles in the exhalation and in the stabilization of the spine is remarkable [9]. Previous studies have shown that subjects with lumbar disorders have lower activity of the abdominal muscles [13], and others also reported a relationship between CLBP and wrong breathing patterns involving the diaphragm and the abdominal muscles [10]. Several investigations also showed different activations and changes in the thickness of abdominal muscles with different breathings [14,15]. In addition, the abdominal muscle thickness has been also studied during exercises which include breathing, such as hypopressive exercises [16] or Pilates [17] and in different positions [18,19]. Nevertheless, these studies did not consider subjects with lumbar disorders and thus the effects of CLBP on the thickness of abdominal muscles appears to be unclear. In this context, a recent investigation analyzed the activity of the abdominal muscles during breathing with ultrasonography in males with and without CLBP [20]. Findings of this investigation suggested that abdominal muscles change differently in males with and without CLBP, with less activity of the external oblique (EO) and greater activity of the rectus abdominis (RA). However, this study does not provide information about the rest condition of the subjects, since only analyze the thickness during breathing, and the sample does not include women, who have been demonstrated to suffer from CLBP more than men [2]. Therefore, the aim of this study is to analyze the differences in the thickness of abdominal muscles at rest and during breathing between subjects of both sexes with and without CLBP.

## 2. Materials and Methods

### 2.1. Study Design and Participants

A cross-sectional study was conducted in order to analyze the differences in the thickness of abdominal muscles between subjects with and without CLBP. The sample size was calculated with the G*Power software (version 3.1, HHU, Düsseldorf, Germany), with a power of 0.95 and an α error of 0.05. Data from Arab et al. [20] were selected, using the variable “thickness at the end of expiration” in the internal oblique (IO) and comparing the groups with (9.53 ± 1.35 mm) and without (8.35 ± 1.63 mm) CLBP. A sample of 20 subjects was estimated as the minimum per group to obtain consistent results.

Subjects were recruited voluntarily from the Faculty of Physiotherapy, in Pontevedra, from September to December, 2019. For the CLBP group, the inclusion criteria were a history of low back pain for at least 3 months, with an intensity from 3 to 5 in a visual analogue scale and with no medication in the last 3 days [8]. For both groups, the exclusion criteria were acute or sub-acute injuries, recent abdominal surgeries, pregnancy and neurological or breathing disorders [8]. All these criteria were analyzed and applied by a physical therapist with knowledge and expertise in the field. Finally, a total of 72 subjects were included and divided in two groups: subjects with CLBP (*n* = 36) and subjects without CLBP (*n* = 36). All participants received oral and written information about the study and signed a written informed consent. Ethical approval was granted by the Ethics Committee of the Faculty of Physiotherapy (University of Vigo) with the code 17072018. The principles of the Declaration of Helsinki were followed.

### 2.2. Procedures

The muscle thickness was measured in the four abdominal muscles: transversus abdominis (TrA), internal oblique (IO), external oblique (EO) and rectus abdominis (RA) with ultrasound imaging. Specifically, a 5–10 MHz lineal b-model ultrasound transduce was used (SonoSite M-Turbo, SonoSite M-Turbo^®^; FUJIFILM, Bothell, Washington, DC, USA). All participants were lying in supine position, with hips and knees bent, and the physical therapist was located on the dominant side of the subject. In order to measure RA, the probe was located on the anterior area of abdominal wall, laterally to the navel [21]. For TrA, IO and EO, the probe was located laterally between the iliac crest and rib cage [21]. Once the muscle was located, the image was frozen and saved in order to perform later measurements of the thickness. These measurements were conducted using the on-screen Calipper, perpendicular to the hyper-echoic area of the muscle and between fascial borders, in centimeters [22].

A physical therapist, with knowledge and expertise in measuring abdominal muscles with ultrasound imaging, carried out the analysis of the abdominal thickness in two moments: at rest and at the end of a maximal breathing. The maximal breathing consisted of a deep inspiration followed by a full expiration, where the thickness were measured. These two measurements were recorded in randomized order and 5 min were waited between them in order to avoid any influence of the previous situation.

### 2.3. Statistical Analysis

All analyses were conducted with the statistical package SPSS for Macintosh (version 25.0. Armonk, NY, USA: IBM Corp). The normality of the distribution was checked both graphically and using the Kolmogorov–Smirnov test. All results were presented by mean ± standard deviation (SD). A two-way analysis of variance (ANOVA) was carried out to analyze the differences in muscle thickness with two intra-subject factors: low back pain (yes or no) and situation (rest or breathing). Partial eta squared (np^2^) effects sizes were calculated for this analysis. A value of np^2^ ≥ 0.01 indicates a small effect, ≥0.059 indicates a medium effect and ≥ 0.138 indicates a large effect [23]. Moreover, pair-wise comparisons between both groups at rest and at the end of the breathing were conducted via Bonferroni post-hoc test, using Cohen’s d to calculate the effect size [23]. These effects were classified as trivial (d < 0.2) small (0.2 < d < 0.5), medium (0.5 < d < 0.8), and large (d ≥ 0.8). For all analysis, the significance value was set at *p* ≤ 0.05.

## 3. Results

In total, 72 subjects were included in this study (64% women), and were divided in two groups: subjects with CLBP (*n* = 36) and control group (*n* = 36) of subjects without CLBP. Descriptive data of participants for both groups are detailed in Table 1.

Comparing descriptive data detailed in Table 1, there were no significant differences in any anthropometric variable between groups (*p* > 0.05).

Results of the comparison of the muscle thickness between subjects with and without CLBP are detailed in Table 2, analyzing also the differences at rest and during breathing.

In TrA, there were no significant differences in muscle thickness between subjects with and without low back pain, neither at rest (*p* = 0.547) nor during breathing (*p* = 0.277). Moreover, muscle thickness increased significantly during breathing in comparison with rest in both groups, but a higher effect has been described in subjects with CLBP (*p* < 0.001; d = 2.35, large) in comparison with the control group (*p* < 0.001; d = 1.93, large).

Results in IO showed no significant differences in muscle thickness between subjects with and without low back pain, neither at rest (*p* = 0.176) nor during breathing (*p* = 0.847). In this sense, the muscle thickness in IO increased significantly during breathing in comparison with rest in both groups, but with a higher effect on the CLBP group (*p* < 0.001; d = 1.27, large) in comparison with subjects in the control group (*p* < 0.001; d = 0.97, large).

In the muscle thickness of EO, no significant differences were found at rest (*p* = 0.613) or during breathing (*p* = 0.403). Besides, no significant changes were reported comparing muscle thickness at rest and during breathing, neither in the CLBP group (*p* = 0.339; d = 0.14, trivial) nor in the control group (*p* = 0.193; d = 0.3, small).

In RA, there were no significant differences in muscle thickness between subjects with and without low back pain at rest (*p* = 0.563). However, during breathing subjects with CLBP reported less muscle thickness than subjects from the control group (*p* = 0.042, np^2^ = 0.52, large). In this muscle, thickness appeared not to be influenced by breathing neither in the CLBP group (*p* = 0.547; d = 0.19, trivial) nor in the control group (*p* = 0.402; d = 0.17, trivial).

## 4. Discussion

The aim of this study was to analyze the differences in abdominal muscle thickness between subjects with and without CLBP, at rest and also during breathing. The main results suggest that in TrA and IO, there are no differences between groups in muscle thickness, but those subjects with CLBP increased the muscle thickness during breathing more than participants without pain. Conversely, in EO there were no differences in muscle thickness between groups, and no significant changes were found between rest and breathing. In RA, subjects with CLBP reported less muscle thickness than subjects without pain during breathing, but no changes were found at rest.

Prior studies have reported the effects of breathing in the abdominal muscles’ thickness, with similar findings in TrA and IO but different results in RA and EO [14,15,19,24,25], which suggest different activations probably due to the position. Moreover, a recent study analyzed the abdominal muscle thickness during breathing in subjects with and without CLBP [20], reporting thicker TrA and EO and greater IO and RA in subjects with pain. These results are contrary to our findings in subjects with CLBP, suggesting that TrA and IO increased the muscle thickness during breathing, but RA decreased its muscle thickness in these subjects with pain. These different results could be due to differences in measuring, in the type of breathing or in the sample size, but the most remarkable difference is that Arab et al. [20] only included males in their sample. CLBP has been reported as a prevalent disfunction, with a higher incidence in women in comparison with men [1,2,26]. Furthermore, the physiological and anatomical differences between sexes need to be consider [27], including differences in pelvic floor muscles, in the viscera and in the hip bones [28,29].

On the other hand, the abdominal muscles appear to have similar thickness in subjects with and without CLBP. However, the effects of breathing appear to be different between these two groups. Specifically, in subjects with pain the deepest abdominal muscles, TrA and IO, increase the thickness during breathing more than subjects without pain, suggesting a higher influence of breathing in subjects with CLBP. A recent study reported that CLBP influence lumbar and pelvic muscles, and could create an instability in lumbopelvic area [30]. This investigation conclude that abdominal muscles had more difficulties to stabilize the spine in subjects with pain, in comparison with a control group. Moreover, other studies have demonstrated that subjects with CLBP had lower activity of the diaphragm during breathing, in comparison with healthy subjects [10,13,31]. Considering also that during breathing the abdominal muscles increase their activation [32,33], subjects with CLBP could need a higher increase of muscle thickness in the deepest abdominal muscles to stabilize the spine and perform a correct breath pattern, in comparison with healthy subjects.

In this sense, the alteration of the breathing patterns has been also reported in subjects with CLBP, influencing the diaphragm but also the abdominal muscles [10,12,13]. The closest disposition of this muscles and the clear relationship between them [11,30], especially with RA, could explain the significant decrease of the thickness of RA during breathing in subjects with CLBP, finding a lower activation of this muscle associated to a probable wrong breath pattern. Nevertheless, this hypothesis has not been tested yet, neither in this study nor in other similar investigations.

Bearing in mind the results mentioned, some limitations have to be considered. First, the thickness of abdominal muscles and the consequent activation during breathing is conditional on the anthropometric characteristics of the subjects, especially on the BMI, and also on the age or the physical activity [28]. Therefore, results in participants with other anthropometric variables or other age range could be different. Second, CLBP has been reported as a global problem involving the spine and the musculoskeletal system, but subjects with specific dysfunctions in this area such as hernias or degeneration of the vertebras could have different muscle thickness. Third, ultrasound imaging has been demonstrated to be a valid and reliable method to assess muscle thickness [34,35,36,37,38]. However, the activation of the muscle when muscle thickness increases needs to be confirmed by other methods, such as electromyography. Besides, only the dominant side of the abdominal muscles was recorded.

Future research should combine ultrasound imaging and electromyography to provide more consistent results, in samples of both sexes and including different age ranges. Moreover, analyzing the breathing pattern could be of great interest to understand the relationship between this and the behavior of abdominal muscles in subjects with CLBP.

## 5. Conclusions

Abdominal muscles have similar muscle thickness at rest in subjects with and without CLBP, but the effects of breathing, finding an increase of the thickness in TrA and IO and a decrease in RA in subjects with CLBP.

This study provides novel information about the behavior of the abdominal muscles in subjects with CLBP in different situations, helping health care and clinical professionals to understand better the influence of these muscles in the CLBP and their implications in rehabilitation.

## Figures and Tables

**Table 1:** Descriptive data of the participants for both groups.

Variables	CLBP Group (mean ± SD)	Control Group (mean ± SD)	*p*-Value
Age (years)	22.44 ± 3.05	21.97 ± 4.23	0.589
Weight (kg)	63.87 ± 11.05	67.07 ± 10.27	0.207
Height (cm)	167.03 ± 7.75	170.39 ± 9.37	0.102
BMI	22.81 ± 1.97	23.01 ± 2.07	0.799

BMI: body mass index; CLBP: chronic low back pain; SD: standard deviation.

**Table 2:** Comparison of the muscle thickness between groups at rest and during breathing.

Group	TrA		IO		EO		RA	
	Rest	Breath	Rest	Breath	Rest	Breath	Rest	Breath
CLBP (cm)	0.31 ± 0.09	0.69 ± 0.21 *	0.72 ± 0.27	1.23 ± 0.55 *	0.6 ± 0.21	0.57 ± 0.22	1.03 ± 0.23	0.99 ± 0.18
Control (cm)	0.33 ± 0.11	0.63 ± 0.22 *	0.81 ± 0.29	1.26 ± 0.59 *	0.58 ± 0.18	0.53 ± 0.15	1.06 ± 0.21	1.1 ± 0.24 #

CLBP: chronic low back pain; TrA: transversus abdominis; IO: internal oblique; EO: external oblique; RA: rectus abdominis. * Significant differences between rest and breathing (*p* < 0.001), # Significant differences between CLBP and control group (*p* < 0.05).
